# Resolution of pleura-peritoneal fistula via transient daytime ambulatory peritoneal dialysis regime (DAPD) – 8 years follow up

**DOI:** 10.12669/pjms.325.11096

**Published:** 2016

**Authors:** Thiam Seong Christopher Lim, Kah Mean Thong

**Affiliations:** 1 Thiam Seong Christopher Lim, Nephrology Unit, Department of Medicine, University Putra Malaysia, Serdang, Malaysia; 2 Kah Mean Thong, Department of Medicine, Ipoh Hospital, Perak, Malaysia

**Keywords:** Pleuro-peritoneal fistula, Peritoneal dialysis, End stage renal failure

## Abstract

Pleural effusion or hydrothorax is a relatively rare but well-recognized complication associated with peritoneal dialysis (PD). We describe the successful long term resolution of a patient who developed pleural effusions after starting continuous ambulatory peritoneal dialysis (CAPD), by altering the PD prescription to normal volume daytime ambulatory peritoneal dialysis (DAPD) transiently before resuming the usual CAPD exchanges four months later. After 8 years of follow up, there is no sign of recurrence of the effusion. Normal volume DAPD present as an attractive alternative and cheap method for resolution of pleura-peritoneal fistula.

## INTRODUCTION

Pleura-peritoneal fistula is a relatively rare but well-recognized complication associated with peritoneal dialysis (PD). This condition although not life-threatening, poses management dilemma to the attending nephrologist. Pleura-peritoneal leak usually requires surgical intervention and despite that recurrence is high and it frequently leads to discontinuance of PD treatment in majority of patients. We describe the successful management of a patient who developed pleural effusions after starting continuous ambulatory peritoneal dialysis (CAPD), by altering the PD prescription to daytime ambulatory peritoneal dialysis (DAPD) transiently before resuming CAPD 4 months later.

## CASE REPORT

The patient was a 53 year-old Punjabi man with hypertension, hepatitis C and end stage renal disease (ESRD) of unknown cause opted for CAPD. During his follow-up, he was noted to have poor effluent outflow with retention of dialysate about 50 to 700ml per day with progressive dyspnoea.

**Fig.1:**
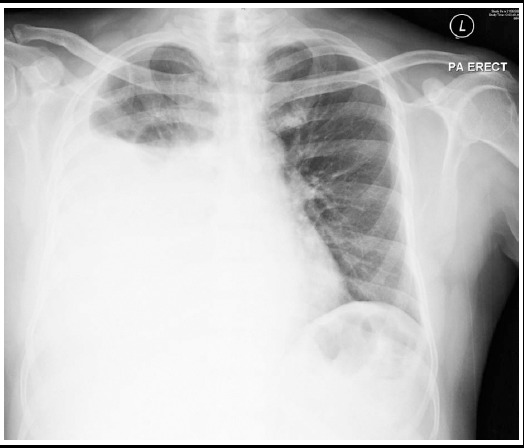
Chest X-ray shows significant right pleural effusion.

**Fig.2:**
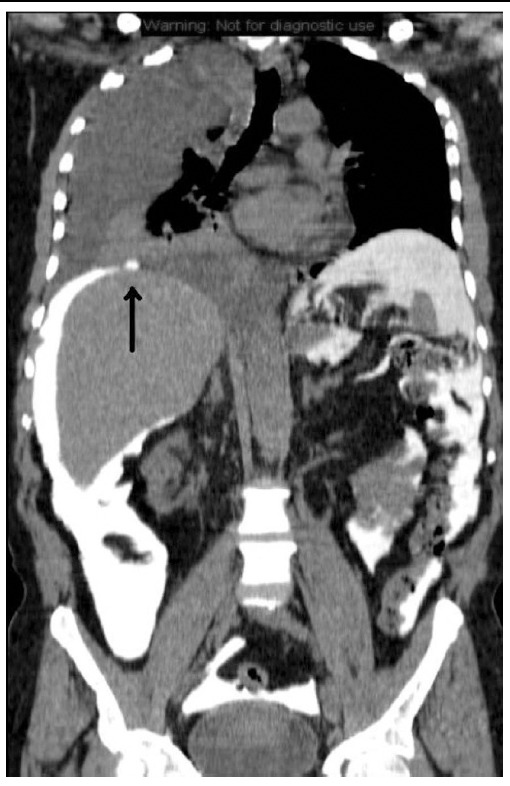
Computed tomography of the thorax shows small contrast filled bleb like lesion in right posterior diaphragm (arrowed).

Clinical examination and chest X-ray disclosed a significant right pleural effusion (Fig.1). Diagnosis of pleuro-peritoneal fistula was made after thoracocentesis results showed high aspirate glucose level compare to plasma glucose level. A contrasted computed tomography of the thorax and abdomen showed bleb-like lesion seen in the right posterior diaphragm (Fig.2) which is highly suggestive of the presence of pleuro-peritoneal fistula.

As the patient had vascular access problem and reluctant to the idea of temporary dialysis catheter, treatment with PD was continued with change of dialysis prescription to DAPD. Patient was advised to do 4 exchanges during daytime by using dialysate volume of 2 litres, and keeping night time “dry”. Serial chest X-rays showed that the effusion was progressively getting smaller and complete re-absorption was seen four months later. Patient was resumed on CAPD with 2L dialysate exchanges four times per day, without recurrence of pleural effusion for the subsequent 8 years of follow-up.

## DISCUSSION

PD related pleural effusion is an important complication to be recognized and treated.^1^ Causes of pleural effusion secondary to other causes such as fluid overload and para-pneumonic effusion will have to be ruled out quickly. Very few nephrologists will choose to continue PD treatment while the patient has persistent leaking of dialysate. The usual management is cessation of PD and conversion to temporary haemodialysis for 2-4 weeks.^2^,^3^ In order to avoid the inconvenience of changing to heamodialysis, some authors dialyze patients by using low volume intermittent PD;^4^ or Automated PD at night with icodextrin at daytime.^5^ Use of hypertonic solutions with “night time rest” had been described as well.^5^ Patients with recurrent pleural effusion can be managed by pleurodesis with tetracycline, talc or autologous blood.^6^ Diagnostic and therapeutic video-assisted thoracoscopy (VATS) allows identification and closure of any defects in the diaphragm.^7^ This procedure is generally more preferred than the invasive surgical repair of the pleuroperitoneal fistula via thoracotomy. However, despite all these interventional therapies, only 50-58% of patients are able to resume long term PD.^2^,^3^

There is evidence that continuation of PD may be beneficial as the dialysate itself could act as a sclerosant by promoting “sealing” of the epithelial layers.^8^ In this case we simply convert the patient to transient DAPD without major disruption in the dialysis modality.

We believe this is the first case report of successful long term resolution of pleura-peritoneal fistula with normal volume DAPD. Our experience with DAPD, which consists of multiple short dwell, normal volume daytime exchanges for treatment of pleura-peritoneal effusion is truly encouraging. Besides providing a natural sealant for the epithelial layers, this treatment aims to reduce the pressure gradient between the pleural and peritoneal cavity that could perpetuate the leaking. Although this method considerably shortens the dialysis exchange time, we found that the clearance of fluid and toxin are not significantly altered as the patient was a newly diagnosed ESRD patient with good residual urine output. The patient was promptly switched back to CAPD after four months and it is worthwhile to note that the patient has no recurrence of the pleural effusion even after 8 years of follow up.

In this case we have shown that by using DAPD, we have managed to avoid the cumbersome method of cessation of PD with conversion to haemodialysis. Furthermore, we also avoided any possible invasive surgical intervention which carries with it certain morbidities.

## References

[1] Nomoto Y, Suga T, Nakajima K, Sakai H, Osawa G, Ota K (1989). Acute hydrothorax in continuous ambulatory peritoneal dialysis--a collaborative study of 161 centers. Am J Nephrol.

[2] Lew SQ (2010). Hydrothorax:pleural effusion associated with peritoneal dialysis. Perit Dial Int.

[3] Ramon RG, Carrasco AM (1998). Hydrothorax in peritoneal dialysis. Perit Dial Int.

[4] Girault-Lataste A, Abaza M, Valentin J (2004). Small volume APD as alternative treatment for peritoneal leaks. Perit Dial Int.

[5] Christidou F, Vayonas G (1995). Recurrent acute hydrothorax in a CAPD patient:successful management with small volumes of dialysate. Perit Dial Int.

[6] Hidai H, Takatsu S, Chiba T (1989). Intrathoracic instillation of autologous blood in treating massive hydrothorax following CAPD. Perit Dialy Int.

[7] Lang CL, Kao TW, Lee CM, Tsai CW, Wu MS (2008). Video-assisted thoracoscopic surgery in continuous ambulatory peritoneal dialysis-related hydrothorax. Kidney Int.

[8] Shemin D, Clark DD, Chazan JA (1989). Unexplained pleural effusions in the peritoneal dialysis population. Perit Dial Int.

